# Glycation-Driven Mitochondrial and ER Stress Underlies Iodoacetic Acid-Induced Apoptosis in Porcine Uterus and Oviduct Epithelial Cells

**DOI:** 10.3390/antiox15050545

**Published:** 2026-04-25

**Authors:** Qin-Yue Lu, Ying-Yan Jin, Cheng-Lin Zhan, Song-Hee Lee, Ji-Yeon Lee, Xiang-Shun Cui

**Affiliations:** Department of Animal Science, Chungbuk National University, Cheongju 28644, Republic of Korea; surpassway@gmail.com (Q.-Y.L.); ehrous86@gmail.com (Y.-Y.J.); zcl@chungbuk.ac.kr (C.-L.Z.); son9heee@gmail.com (S.-H.L.); dlwldus2033@chungbuk.ac.kr (J.-Y.L.)

**Keywords:** iodoacetic acid, reproductive epithelial toxicity, glycational stress, mitochondrial dysfunction, calcium homeostasis

## Abstract

Iodoacetic acid (IAA), a highly cytotoxic disinfection byproduct commonly detected in drinking water, poses a potential risk to female reproductive health. The direct molecular mechanisms underlying its effects on the reproductive system epithelium remain unclear. This study demonstrates that IAA induces glycational stress in primary porcine uterine (UECs) and oviduct epithelial cells (OECs), representing an early event contributing to extensive cellular toxicity. IAA exposure inhibited Glyceraldehyde-3-Phosphate Dehydrogenase (GAPDH) enzymatic activity and promoted the accumulation of advanced glycation end products (AGEs) Nε-(carboxymethyl)lysine (CML), triggering mitochondrial dysfunction, redox imbalance, calcium dyshomeostasis, and endoplasmic reticulum stress. These disturbances activated a dysregulated signaling network involving the p38 MAPK, AKT, and NF-κB pathways, ultimately causing G1/S cell cycle arrest and apoptosis. Notably, pretreatment with the AGE inhibitor pyridoxamine reduced CML accumulation, restored mitochondrial function, and alleviated apoptotic cell death. These findings identify glycational stress as a key initiating mechanism for IAA-induced reproductive epithelial toxicity, providing mechanistic insight into the potential health risks of environmental disinfection byproducts.

## 1. Introduction

Iodoacetic acid (IAA) is a highly cytotoxic haloacetic acid formed as a disinfection byproduct during the chlorination of drinking water [1,2,3,4,5]. Extensive toxicological investigations have demonstrated that IAA induces oxidative stress, mitochondrial dysfunction, and DNA damage in various biological systems [3]. Within the reproductive system, accumulating evidence indicates that IAA disrupts ovarian function, impairs oocyte maturation, and promotes granulosa cell apoptosis, collectively posing a potential risk to female fertility [6,7,8,9]. Recent studies suggest that IAA exposure may interfere with key steps in steroidogenesis, including the expression and activity of enzymes involved in estradiol and progesterone biosynthesis, thereby disturbing hormonal signaling between granulosa cells, theca cells, and the oocyte [10,11,12].

In addition to endocrine imbalance, oxidative stress and mitochondrial injury represent central pathological features associated with IAA exposure [13]. Granulosa cells and oocytes rely on mitochondrial ATP production to sustain meiotic spindle assembly, chromatin organization, and epigenetic stability. Thus, elevated reactive oxygen species and loss of mitochondrial membrane potential disproportionately weaken their survival and developmental potential. Although these cellular consequences have been observed, the sequential molecular events linking oxidative injury, metabolic stress, inflammatory signaling, and programmed cell death under IAA exposure have not yet been systematically delineated. Consequently, the initiating triggers and the full mechanistic cascade underlying IAA-induced reproductive cytotoxicity remain incompletely understood. Furthermore, the impact of IAA on the epithelial lining of the uterus and oviduct, which serves as the first line of defense and functional interface of the female reproductive tract, remains largely unexplored.

The female reproductive system relies on the coordinated function of multiple anatomical structures, among which the uterus and oviduct are fundamental to reproductive success [14,15]. Within this context, uterine epithelial cells (UECs) and oviduct epithelial cells (OECs) play indispensable roles. UECs and OECs together form the first line of defense, regulatory interface, and structural foundation for reproductive competence. Given their constant exposure to luminal fluids, environmental chemicals, and endocrine signals, these epithelial tissues are particularly vulnerable to toxic insults [16]. Therefore, evaluating how environmental contaminants directly affect UECs and OECs is critical for understanding the cellular basis of reproductive toxicity. One potential mechanism linking chemical exposure to epithelial injury is glycational stress.

Glycational stress refers to a pathological condition characterized by the excessive formation and accumulation of advanced glycation end products (AGEs) [17]. The accumulation of AGEs is widely recognized to impair protein stability and enzymatic activity, disrupt cytoskeletal dynamics, and promote protein misfolding. In addition, AGEs stimulate the production of reactive oxygen species and activate pro-inflammatory signaling cascades, ultimately contributing to mitochondrial dysfunction, redox imbalance, and tissue injury [18]. While glycational stress is most commonly associated with hyperglycemia and diabetes, accumulating evidence indicates that exogenous chemical agents can also trigger or accelerate this process.

Although it is most commonly associated with glucose-derived carbonyl compounds, increasing evidence indicates that exogenous chemical agents can also accelerate AGE formation [19,20]. Such agents may introduce reactive electrophilic species, alter cellular redox buffering capacity, or interfere with metabolic enzyme function, thereby increasing the availability of carbonyl intermediates that participate in non-enzymatic glycation reactions [21,22,23]. In the present study, IAA exposure led to cellular phenotypes that closely mirror the known consequences of glycational stress, including metabolic disruption, mitochondrial structural collapse, heightened reactive oxygen species production, and activation of inflammatory signaling pathways. These parallels suggest that IAA may exert its cytotoxic effects, at least in part, by promoting a glycational stress-like state, thereby initiating a cascade of downstream pathological events.

Based on this hypothesis, this study investigated whether IAA induces reproductive epithelial toxicity through glycational stress. Primary porcine UECs and OECs were selected as in vitro models of the reproductive system epithelial barrier. The occurrence of glycational stress following IAA exposure was confirmed by evaluating GAPDH enzymatic activity and the accumulation of AGEs, with CML serving as a representative marker. Subsequently, alterations in cellular metabolism, redox balance, calcium homeostasis, mitochondrial function, and the activation of stress and inflammatory signaling pathways were systematically analyzed to delineate the mechanistic cascade from initial molecular events to resulting cell fate outcomes. The causal contribution of glycational stress was further validated through rescue experiments using the AGE inhibitor pyridoxamine. Overall, this study provides evidence for a potential mechanism in which IAA compromises the female reproductive epithelium by inducing glycational stress, thereby providing mechanistic insights and molecular targets for evaluating the reproductive health risks associated with disinfection byproducts. The specific knowledge gap addressed herein is twofold: first, the early molecular events that couple IAA exposure to mitochondrial dysfunction and apoptosis in reproductive tract epithelia have not been systematically delineated; second, the potential role of glycative stress as an upstream driver in this cascade remains untested.

## 2. Materials and Methods

### 2.1. Ethics Statement

The present study did not involve any live animals. All tissues used in this study were obtained from a licensed commercial slaughterhouse as by-products of routine meat production. The animals were slaughtered for food purposes and were not sacrificed or handled specifically for research. Therefore, no ethical approval was required for this study in accordance with the institutional and national guidelines.

### 2.2. Primary Cell Isolation and Culture

All procedures involving porcine tissues were conducted in accordance with the Institutional Animal Care and Use Committee (IACUC) guidelines of the Chungbuk National University Laboratory Animal Center (Cheongju, South Korea).

Porcine uterine epithelial cells (UECs) and porcine oviduct epithelial cells (OECs) were isolated using enzymatic dissociation. Briefly, tissues were rinsed in phosphate-buffered saline and digested with a collagenase solution containing Collagenase IV (17104019, Thermo, Waltham, MA, USA) and Collagenase Type II (17101015, Thermo). The resulting cell suspensions were filtered, centrifuged, and plated in growth medium.

Cells were maintained in DMEM/F-12 medium (11320033, Thermo, Waltham, MA, USA) supplemented with 10% fetal bovine serum (FBS, 12483020, Thermo, Waltham, MA, USA) and 1% penicillin–streptomycin (15140148, Thermo, Waltham, MA, USA) at 37 °C in a humidified atmosphere containing 5% CO_2_. Epithelial identity was verified by immunofluorescence staining for Cytokeratin 7 (CK7, 4465T, Cell Signaling Technology, Danvers, MA, USA).

### 2.3. Chemical Treatments

Iodoacetic acid (IAA; I4386, Sigma, St. Louis, MI, USA) was dissolved in sterile water to prepare stock solutions and diluted in culture medium to the indicated final concentrations. Unless otherwise specified, cells were exposed to IAA for 24 h prior to subsequent analyses. For rescue experiments, cells were pre-treated with pyridoxamine (P9158, Sigma, St. Louis, MI, USA; 1 mM final concentration in culture medium) for 2 h before the addition of IAA, after which cells were co-incubated with both pyridoxamine and IAA for 24 h.

### 2.4. Cell Viability Assay

Cell viability was evaluated using the Cell Counting Kit-8 assay (C0038, Beyotime, Shanghai, China) following the manufacturer’s protocol. Briefly, cells were seeded into 96-well plates and allowed to adhere under standard culture conditions. After attachment, cells were treated with graded concentrations (0, 0.02, 0.04, 0.1, 0.2, 0.5, 0.75, 1, 2, and 5 μM) of IAA for 24 h. Subsequently, CCK-8 reagent was added to each well and incubated for 2 h, and absorbance at 450 nm was measured using a microplate reader. The half-maximal inhibitory concentration (IC_50_) was calculated by fitting the dose–response curve using nonlinear regression analysis. Each experiment included a vehicle control group (untreated cells cultured in complete medium containing an equivalent volume of sterile water) and IAA-treated groups at the indicated concentrations. All assays were performed in three independent biological replicates, with each replicate comprising six technical wells per condition.

### 2.5. Wound Healing Assay

Wound healing assay was performed to assess cell migration. Briefly, cells were seeded into 6-well plates and cultured until a confluent monolayer was formed. A linear scratch was generated across the monolayer using a sterile 200 µL pipette tip, followed by gentle washing with PBS to remove detached cells. Fresh serum-reduced medium was then added to minimize proliferation-driven closure. Images of the wound area were acquired immediately (0 h) and after 12 h of incubation using an inverted microscope. The wound width was measured at multiple predefined points, and the percentage of wound closure was quantified using ImageJ software (1.54p). Three independent biological replicates were performed for each cell type.

### 2.6. Cell Cycle Analysis

Cell cycle distribution was evaluated using propidium iodide (PI) staining followed by flow cytometry. Cells were collected, washed with PBS, and fixed in 70% ice-cold ethanol at 4 °C overnight. Fixed cells were processed using the Cell Cycle and Apoptosis Analysis Kit (C1052, Beyotime, Shanghai, China) according to the manufacturer’s protocol. Briefly, samples were incubated with RNase A and stained with PI in the dark at room temperature. Flow cytometric acquisition was performed on a FACSymphony A3 flow cytometer (BD Biosciences, Franklin Lakes, NJ, USA). Cell population percentages in the G0/G1, S, and G2/M phases were quantified using FlowJo software (v10.9.0). For each cell type, three independent biological replicates were analyzed. Each replicate included the vehicle control group and IAA-treated group. Flow cytometric acquisition was performed on three samples per condition per replicate.

### 2.7. Apoptosis Assay

Apoptotic cell death was quantified using the Annexin V-FITC/PI Apoptosis Detection Kit (V13242, Thermo, Waltham, MA, USA) following the manufacturer’s instructions. Briefly, cells were harvested, washed twice with cold PBS, and resuspended in binding buffer. Annexin V-FITC and PI were added, and samples were incubated in the dark at room temperature for the recommended duration. Flow cytometric acquisition was performed on a FACSymphony A3 flow cytometer (BD Biosciences). The proportions of viable, early apoptotic, and late apoptotic/necrotic cells were analyzed using FlowJo software (v10.9.0). For each cell type, three independent biological replicates were analyzed. Each replicate included the vehicle control group and IAA-treated group. Flow cytometric acquisition was performed on three samples per condition per replicate.

### 2.8. Quantitative Reverse Transcription-Polymerase Chain Reaction (qRT-PCR)

Total RNA was extracted using TRIzol reagent (T3934, Sigma, St. Louis, MI, USA) following the manufacturer’s protocol. RNA concentration and purity were evaluated using a microvolume spectrophotometer, and RNA integrity was verified by agarose gel electrophoresis. Reverse transcription was performed using 2X AmpMasterTaq (GeneAll, 541-050, Suwon, Gyeonggi-do, Republic of Korea). Quantitative PCR analysis was conducted using RealAmp SYBR qPCR Master Mix (GeneAll, 801-050, Suwon, Gyeonggi-do, Republic of Korea) on a QuantStudio™ 6 Real-Time PCR System (4485692, Thermo, Waltham, MA, USA). All reactions were performed in triplicate. GAPDH served as the internal control, and relative gene expression levels were calculated using the 2^−ΔΔCT^ method. Primer sequences are listed in Table 1. For each cell type, three independent biological replicates were analyzed. All qPCR reactions were performed in technical triplicate, and the mean threshold cycle (Ct) value was used for subsequent analysis.

### 2.9. Western Blotting

Cells were lysed in RIPA lysis buffer supplemented with protease and phosphatase inhibitors. Protein concentrations were quantified using the Pierce™ BCA Protein Assay Kit (23227, Thermo). Equal amounts of protein were separated by SDS-PAGE and transferred onto PVDF membranes. Membranes were blocked with 5% non-fat milk in TBST for 1 h at room temperature and subsequently incubated overnight at 4 °C with the appropriate primary antibodies. These included: rabbit anti-GAPDH (1:1000, 5174, Cell Signaling Technology), rabbit anti-NF-κB p65 (1:1000, 80979-1-RR, Proteintech, Chicago, IL, USA), and rabbit anti-phospho-p38 MAPK (Thr180/Tyr182) (1:1000, 9211S, Cell Signaling Technology). After primary antibody incubation, membranes were washed three times in 1× TBST (10 min each) and incubated with HRP-conjugated goat anti-rabbit IgG secondary antibodies (sc-2004, Santa Cruz Biotechnology, Dallas, TX, USA) for 1 h at room temperature. Protein bands were visualized using an ECL detection reagent (WP20005, Thermo, Waltham, MA, USA). Densitometric analysis was performed using ImageJ. Protein expression levels were normalized to GAPDH and reported relative to the control conditions. Three independent biological replicates were performed for each cell type, with each replicate containing the vehicle control group and IAA-treated group.

### 2.10. Immunofluorescence

Cells were washed with PBS and fixed in 3.7% paraformaldehyde for 30 min at room temperature, followed by permeabilization with 0.5% Triton X-100 for 30 min. After blocking in 1% bovine serum albumin for 1 h, cells were incubated overnight at 4 °C with primary antibodies diluted in blocking buffer. The primary antibodies included rabbit anti-NRF2 (16396-1-AP, Proteintech, Chicago, IL, USA), rabbit anti-NF-κB p65 (8242, Cell Signaling Technology), and rabbit anti-GRP78/BiP (ab21685, Abcam, Cambridge, UK). After three washes with PBS, cells were incubated for 1 h at 37 °C with fluorophore-conjugated secondary antibodies, 546™ donkey anti-rabbit IgG (H+L) (A10040, Invitrogen, Carlsbad, CA, USA), depending on the primary antibody host species. Hoechst 33342 (5 μg/mL) was used to stain chromatin to enable nuclear visualization. Samples were mounted and examined using a confocal laser scanning microscope (LSM 710 META; Zeiss, Oberkochen, Germany). Image acquisition was performed using Zen software (v8.0), and fluorescence intensity analysis was conducted using ImageJ. For each cell type, three independent biological replicates were performed. Within each replicate, duplicate wells were prepared for the control and IAA-treated groups. A minimum of five randomly selected fields per well were captured for subsequent fluorescence intensity analysis.

### 2.11. Mitochondrial Stress Test

Mitochondrial respiratory function was evaluated using the Seahorse XF Cell Mito Stress Test Kit (103015-100, Agilent, Santa Clara, CA, USA) on a Seahorse XF Pro Analyzer (Agilent Technologies, Santa Clara, CA, USA). Cells were seeded into Seahorse XF cell culture microplates at an optimized density and allowed to adhere overnight. Prior to the assay, cells were washed and incubated in Seahorse XF Assay Medium supplemented with 1 mM pyruvate, 2 mM glutamine, and 10 mM glucose, followed by a 60-min equilibration period in a non-CO_2_ incubator. The oxygen consumption rate (OCR) was recorded under basal conditions and after sequential injections of oligomycin (ATP synthase inhibitor), FCCP (mitochondrial uncoupler), and a mixture of rotenone and antimycin A (complex I and III inhibitors). Basal respiration, ATP-linked respiration, maximal respiration, and spare respiratory capacity were calculated according to the manufacturer’s analysis protocol. Data processing and normalization were performed using Wave software (v2.6) (Agilent Technologies), and the results were expressed relative to the control conditions. Three independent biological replicates were performed for each cell type. Within each replicate, the vehicle control group and IAA-treated group were assayed in parallel using separate wells (six technical replicate wells per condition per replicate). Oxygen consumption rate data were normalized to protein content or cell number as specified in the analysis protocol.

### 2.12. GSH/GSSG Ratio Assay

The cellular redox status was evaluated by determining the ratio of reduced glutathione (GSH) to oxidized glutathione (GSSG) using the GSH/GSSG Assay Kit (S0053, Beyotime). Cells were lysed on ice with the protein removal reagent provided in the kit to prevent non-enzymatic oxidation during sample preparation. The lysates were centrifuged at 10,000× *g* for 10 min at 4 °C, and the supernatants were collected for analysis. Total GSH levels were measured by incubating the samples with the DTNB/NADPH working solution according to the manufacturer’s instructions. For GSSG quantification, GSH was derivatized using the GSH masking reagent prior to detection. Absorbance was measured at 412 nm using a Sunrise™ microplate reader (Tecan, Männedorf, Zurich, Switzerland). The GSH/GSSG ratio was calculated based on the measured concentrations of GSH and GSSG and normalized to the untreated control group. Three independent biological replicates were analyzed for each cell type.

### 2.13. GAPDH Enzyme Activity Assay

GAPDH enzymatic activity was quantified using the GAPDH Activity Assay Kit (MAK277, Sigma) following the manufacturer’s instructions. Briefly, cells were lysed on ice, and the lysates were clarified by centrifugation at 12,000× *g* for 10 min at 4 °C. The supernatants were collected, and protein concentrations were determined using the BCA assay. GAPDH activity was assessed by monitoring the rate of NAD^+^ reduction to NADH at 450 nm in the presence of glyceraldehyde-3-phosphate as the substrate. The increase in absorbance was recorded kinetically using a microplate reader, and enzyme activity was calculated based on the slope of the linear reaction phase. All measurements were normalized to total protein content and expressed relative to the untreated control group. Three independent biological replicates were performed for each cell type. Each replicate included a vehicle control group and IAA-treated group, with enzyme activity measured in technical duplicate.

### 2.14. Quantification of CML

The intracellular level of CML was quantified using the OxiSelect™ Nε-(carboxymethyl) lysine Competitive ELISA Kit (STA-816, Cell Biolabs, San Diego, CA, USA), following the manufacturer’s instructions. Briefly, cells were lysed on ice and centrifuged at 12,000× *g* for 10 min at 4 °C to obtain clarified supernatants. Total protein concentrations were determined using the BCA assay, and equal amounts of protein were applied to the ELISA plate. After sequential incubation with the CML conjugate and primary antibody, the reaction was developed using the supplied chromogenic substrate. Absorbance was measured at 450 nm using a Sunrise™ microplate reader (Tecan, Männedorf, Zurich, Switzerland). CML concentrations were calculated based on a standard curve and normalized to total protein content. Three independent biological replicates were analyzed for each cell type. Each replicate contained a vehicle control group and IAA-treated group, and each sample was measured in technical duplicate according to the manufacturer’s protocol.

### 2.15. Intracellular ROS Measurement

Intracellular reactive oxygen species (ROS) levels were assessed using the cell-permeable fluorescent probe 2′,7′-dichlorodihydrofluorescein diacetate (H2DCFDA; C400, Thermo). Cells were incubated with 10 μM H2DCFDA in serum-free medium for 30 min at 37 °C in the dark, followed by two washes with PBS to remove excess probe. For fluorescence imaging, stained cells were observed using a confocal microscope (LSM 710 META, Zeiss) under identical laser and gain settings across treatment groups. Quantitative fluorescence analysis was performed using FlowJo and ImageJ. For each cell type, three independent biological replicates were performed. Within each replicate, duplicate wells were prepared for the control and IAA-treated groups. A minimum of five randomly selected fields per well were captured for subsequent fluorescence intensity analysis.

### 2.16. Mitochondrial Membrane Potential Assay

Mitochondrial membrane potential was evaluated using the JC-1 fluorescent probe (M34152, Thermo). Cells were incubated with 5 μM JC-1 working solution at 37 °C for 20 min in the dark, followed by two washes with warm PBS to remove unbound dye. Mitochondrial membrane potential was quantified by measuring the ratio of JC-1 aggregates to JC-1 monomers. For flow cytometric analysis, fluorescence was detected using a FACSymphony A3 flow cytometer (BD Biosciences) with excitation. Quantitative analysis was performed using FlowJo. For each cell type, three independent biological replicates were analyzed. Each replicate included the vehicle control group and IAA-treated group. Flow cytometric acquisition was performed on three samples per condition per replicate.

### 2.17. Intracellular and Mitochondrial Ca^2+^ Flux Measurement

Intracellular Ca^2+^ levels were assessed using Fluo-4 AM (F14217, Thermo), and mitochondrial Ca^2+^ levels were measured using Rhod-2 AM (S1062S, Beyotime). Briefly, cells were incubated with Fluo-4 or Rhod-2 in serum-free medium at 37 °C for 30 min in the dark, followed by washing twice with PBS and a 20-min de-esterification period at 37 °C before analysis.

Flow cytometric measurements were performed. Forward scatter and side scatter voltages were adjusted to exclude debris and dead cells. A stable baseline fluorescence signal was collected for 40–50 s. Subsequently, Ca^2+^ influx was induced by the rapid addition of ionomycin (I24222, Thermo), followed by gentle mixing for 5 s, and fluorescence acquisition continued for an additional 3–5 min to record dynamic Ca^2+^ flux. Mean fluorescence intensity was calculated, and Ca^2+^ signals were normalized to baseline values. For each cell type, three independent biological replicates were analyzed. Each replicate included the vehicle control group and IAA-treated group. Flow cytometric acquisition was performed on three samples per condition per replicate.

### 2.18. TUNEL Assay

Apoptotic cells were detected using a TUNEL (TdT-mediated dUTP Nick-End Labeling) Assay Kit (C1086, Beyotime). Briefly, cells were fixed with 4% paraformaldehyde for 20 min at room temperature and permeabilized with 0.1% Triton X-100 for 5 min on ice. After washing with PBS, cells were incubated with the TUNEL reaction mixture at 37 °C for 1 h in the dark. Nuclei were counterstained with Hoechst 33342 (5 μg/mL) for 10 min to allow nuclear visualization. Fluorescent images were acquired using a Zeiss LSM 710 META confocal microscope under identical exposure settings. For each cell type, three independent biological replicates were performed. Within each replicate, duplicate wells were prepared for the control and IAA-treated groups. A minimum of five randomly selected fields per well were captured for subsequent fluorescence intensity analysis.

### 2.19. Statistical Analysis

All experiments were performed with at least three independent replicates. Data are presented as mean ± SEM. Statistical significance was determined using the Student’s *t*-test (for two groups) or one-way ANOVA followed by a post hoc test (for multiple groups) in GraphPad Prism (9.0.0). A value of * *p* < 0.05 was considered statistically significant.

## 3. Results

### 3.1. IAA-Induced Cytotoxicity in UECs and OECs

**Figure 1:**
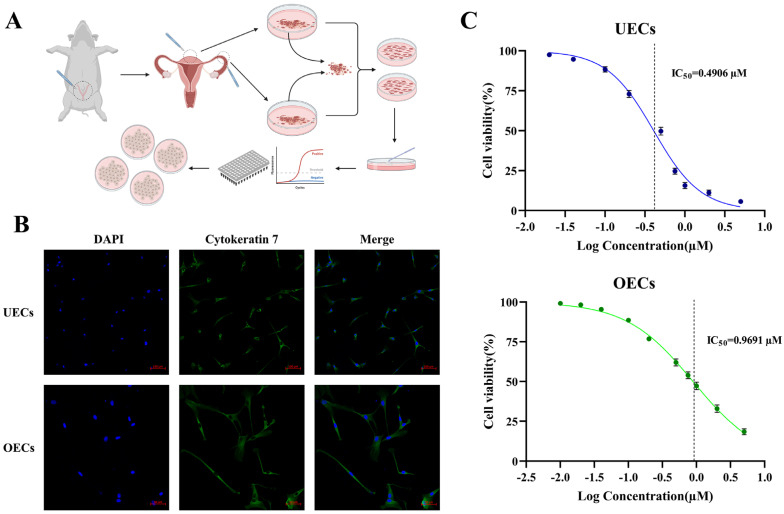
Basic cytotoxic effects of IAA on primary uterine and oviduct epithelial cells. (**A**) Schematic diagram of primary cell isolation and culture. (**B**) Immunofluorescence identification of epithelial cells using cytokeratin 7 (CK7, green), an epithelial cell marker, with nuclei counterstained using DAPI (blue). Scale bar = 100 μm. (**C**) Cell viability of UECs and OECs following IAA exposure and IC_50_ determination. Data are presented as mean ± SEM from three independent biological replicates.

UECs and OECs were isolated and cultured as shown in the schematic diagram (Figure 1A). The epithelial identity of the cultured cells was verified by positive immunostaining for the epithelial marker cytokeratin 7, with nuclei counterstained using DAPI (Figure 1B), confirming the purity and suitability of the models for toxicity evaluation.

The cytotoxic effect of IAA on UECs and OECs was assessed by measuring cell viability following 24-h exposure to increasing concentrations of IAA. A dose-dependent reduction in viability was observed in both cell types (Figure 1C). The calculated half-maximal inhibitory concentrations (IC_50_) were approximately 0.5 µM for UECs and 1 µM for OECs.

### 3.2. IAA Disrupts Redox and Metabolic Balance and Suppresses Migration

**Figure 2:**
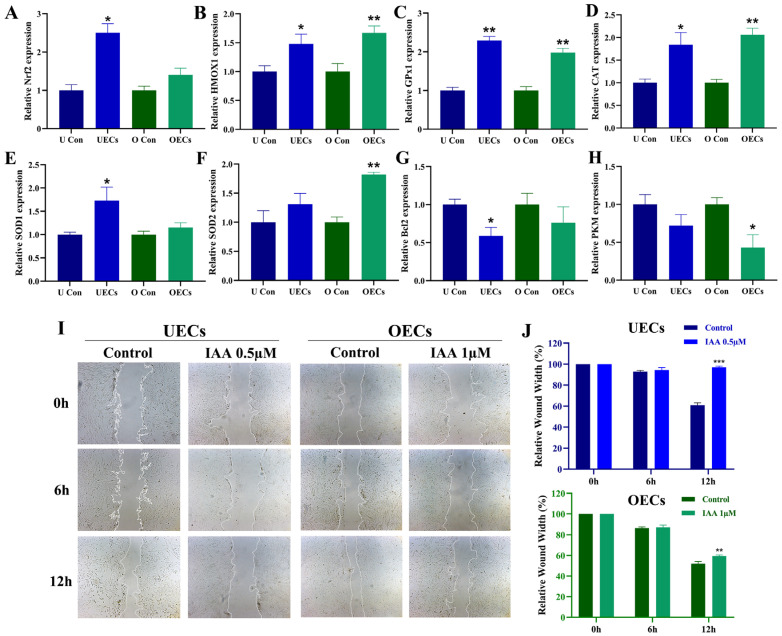
IAA disrupts redox and metabolic balance and migration. (**A**–**H**) qRT-PCR analysis of proliferation-related genes in UECs (IAA 0.5 μM) and OECs (IAA 1 μM). (**A**) NRF2 (nuclear factor erythroid 2-related factor 2). (**B**) HMOX1 (heme oxygenase 1). (**C**) GPX1 (glutathione peroxidase 1). (**D**) CAT (catalase). (**E**) SOD1 (superoxide dismutase 1). (**F**) SOD2 (superoxide dismutase 2). (**G**) BCL2 (B-cell lymphoma 2). (**H**) PKM (pyruvate kinase M). (**I**,**J**) Representative images of wound-healing assay (**I**) and corresponding quantification of wound closure percentage (**J**). Dashed lines indicate the initial wound margins. Wound closure percentage was calculated as [(Area at 0 h − Area at 6/12 h)/Area at 0 h] × 100%. Among them, n = 3, * *p* < 0.05, ** *p* < 0.01, *** *p* < 0.001.

To explore the cellular responses underlying IAA-induced growth inhibition, transcriptional alterations in genes associated with oxidative stress defense and energy metabolism were first examined. Following IAA exposure, distinct changes in the expression of antioxidant and metabolic genes were observed in UECs and OECs (Figure 2A–H). In UECs, the mRNA levels of genes encoding antioxidant enzymes were significantly elevated, including NRF2, HMOX1, GPX1, CAT, and SOD1. In contrast, the anti-apoptotic gene BCL2 was significantly downregulated. In OECs, significant upregulation was observed for HMOX1, GPX1, CAT, and SOD2, while PKM, encoding a key glycolytic enzyme, was significantly downregulated.

To further evaluate the functional consequences of IAA treatment, cell migration and repair capacity were assessed using a wound-healing assay. After 12 h of IAA exposure, the wound closure area was significantly decreased in both UECs and OECs compared with the corresponding controls (Figure 2I,J).

### 3.3. IAA Induces Cell Cycle Arrest and Triggers Apoptosis

**Figure 3:**
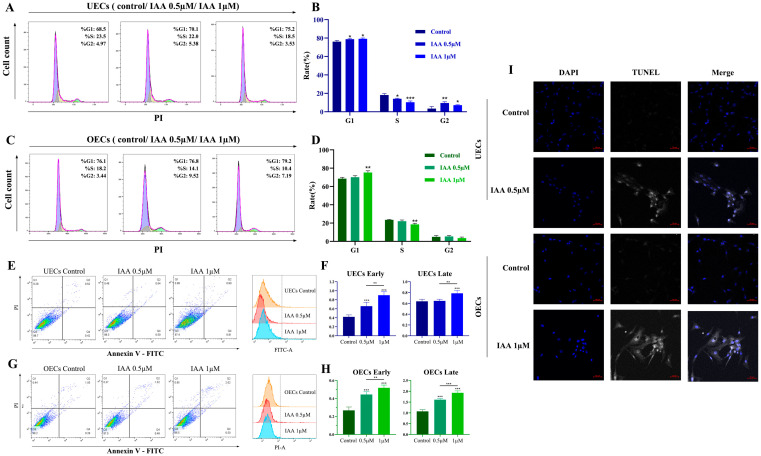
IAA induces cell cycle arrest and apoptosis. (**A**–**D**) Representative flow cytometry histograms (**A**,**C**) and quantification (**B**,**D**) of cell cycle distribution (G1 and S phase percentages). (**E**–**H**) Flow cytometric analysis of apoptosis using Annexin V/PI staining, showing representative plots (**E**,**G**) and quantification (**F**,**H**) of early and late apoptotic cells. (**I**) Representative TUNEL staining. Scale bar = 50 μm. All data are presented as mean ± SEM from three independent biological replicates. * *p* < 0.05, ** *p* < 0.01, *** *p* < 0.001.

To clarify the mechanism underlying the growth-inhibitory effects of IAA, its influence on cell cycle progression and apoptosis was examined. Cell cycle distribution was first analyzed by flow cytometry. IAA exposure led to a marked increase in the proportion of cells in the G1 phase accompanied by a reduction in S-phase cells in both UECs and OECs (Figure 3A–D).

Apoptotic induction was subsequently assessed using Annexin V/PI staining. IAA treatment substantially elevated the proportions of both early and late apoptotic cells (Figure 3E–H). To further confirm apoptosis at the DNA fragmentation level, TUNEL staining was performed. Increased TUNEL-positive signals were observed in IAA-treated cells compared with the controls (Figure 3I).

### 3.4. IAA Impairs Energy Metabolism Redox Status and Calcium Regulation

**Figure 4:**
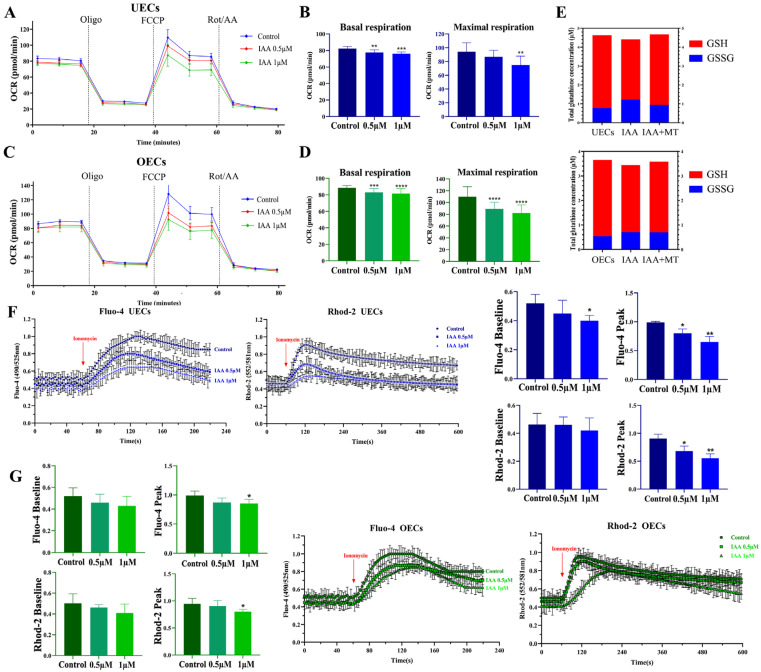
IAA disrupts energy metabolism, redox balance, and calcium homeostasis. (**A**–**D**) Seahorse mitochondrial stress test showing real-time oxygen consumption rate (OCR) curves (**A**,**C**) and quantification of basal and maximal respiration (**B**,**D**). (**E**) Quantification of intracellular GSH/GSSG ratio. (**F**) Cytosolic and mitochondrial Ca^2+^ dynamics in UECs measured by Fluo-4 AM and Rhod-2 AM. Data in bar graphs are presented as mean ± SEM from three independent biological replicates. (**G**) Cytosolic and mitochondrial Ca^2+^ dynamics in OECs measured by Fluo-4 AM and Rhod-2 AM. Data in bar graphs are presented as mean ± SEM from three independent biological replicates. * *p* < 0.05, ** *p* < 0.01, *** *p* < 0.001, **** *p* < 0.0001.

Given that IAA is an inhibitor of glycolytic metabolism, its effects on mitochondrial function, the central hub of cellular energy production, were first examined. Mitochondrial respiration was assessed using the Seahorse mitochondrial stress test. IAA exposure substantially reduced both basal and maximal respiratory capacities in UECs and OECs (Figure 4A–D), indicating pronounced mitochondrial dysfunction and a significant decrease in ATP production via oxidative phosphorylation. Subsequently, intracellular redox balance was evaluated, as glycolytic inhibition and mitochondrial impairment are frequently associated with oxidative stress. A significant decrease in the GSH/GSSG ratio was detected following IAA treatment (Figure 4E), indicating increased oxidative pressure and a breakdown of the antioxidant defense system.

Energy deficiency combined with oxidative stress is known to disrupt intracellular calcium (Ca^2+^) homeostasis. Cytosolic Ca^2+^ dynamics monitored with the Fluo-4 AM probe revealed a substantial disturbance in Ca^2+^ signaling under IAA exposure (Figure 4F,G). Moreover, mitochondrial Ca^2+^ measurements using the Rhod-2 AM probe demonstrated a marked reduction in mitochondrial Ca^2+^ uptake capacity (Figure 4F,G).

### 3.5. IAA Induces Oxidative Stress and Causes Mitochondrial Damage

**Figure 5:**
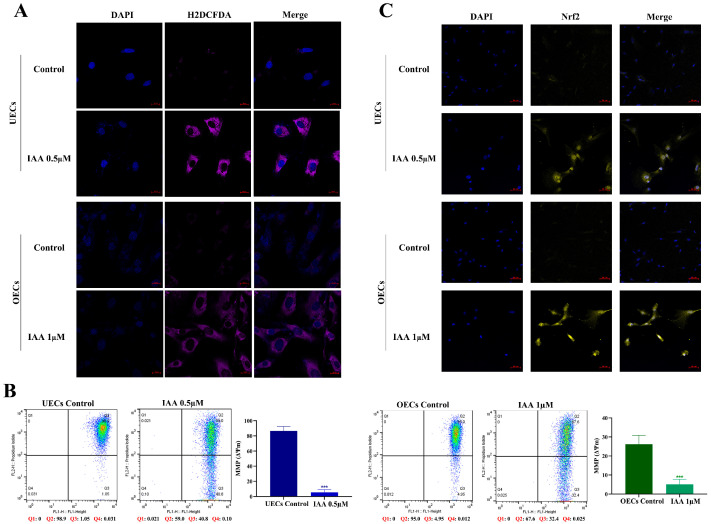
IAA induces oxidative stress and mitochondrial damage. (**A**) Intracellular ROS levels detected by H_2_DCFDA fluorescence with quantification. Scale bar = 20 μm. (**B**) Mitochondrial membrane potential (ΔΨm) assessed by JC-1 staining and flow cytometry. Representative dot plots show JC-1 aggregates, and monomer fluorescence (left), with quantification of the fluorescence ratio (right). (**C**) Subcellular localization of Nrf2 by immunofluorescence. Scale bar = 50 μm. *** *p* < 0.001.

To directly validate that the redox imbalance induced by IAA results in the accumulation of ROS, intracellular ROS levels were assessed using the H2DCFDA fluorescent probe. Cells exposed to IAA exhibited increased green fluorescence signals compared with the control group (Figure 5A).

Excessive ROS is known to contribute to mitochondrial injury. Therefore, the mitochondrial membrane potential was examined using the JC-1 probe. Flow cytometric analysis showed a significant decrease in the JC-1 red/green fluorescence ratio after IAA treatment (Figure 5B).

In response to elevated oxidative stress, cells activate compensatory antioxidant pathways. This adaptive mechanism was evaluated by examining the subcellular localization of the transcription factor *NRF2*. Immunofluorescence staining demonstrated a clear translocation of NRF2 from the cytoplasm into the nucleus following IAA exposure (Figure 5C).

### 3.6. IAA Activates Stress and Inflammatory Signaling Pathways

**Figure 6:**
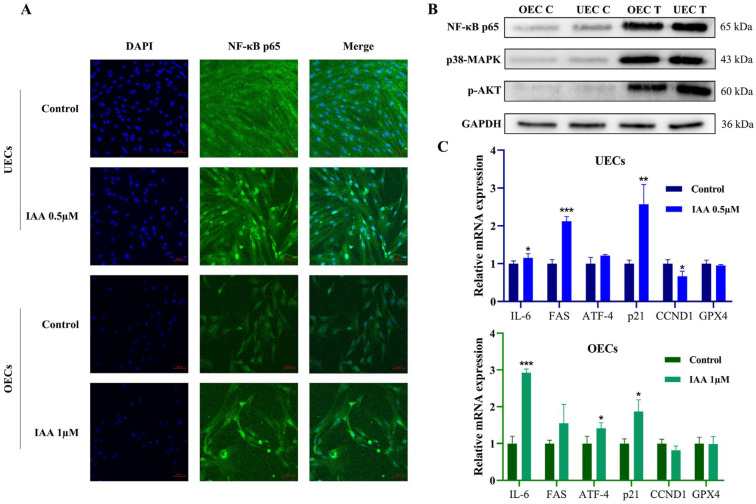
IAA activates stress and inflammatory signaling pathways. (**A**) Immunofluorescence of NF-κB p65 with nuclear counterstaining. Scale bar = 50 μm. (**B**) Representative Western blot bands and quantification of the p38 MAPK, p-AKT, and NF-κB p65 protein levels. (**C**) qRT-PCR analysis of downstream stress and inflammation-related genes. Among them, n = 3, * *p* < 0.05, ** *p* < 0.01, *** *p* < 0.001.

The cellular disturbances described above, including metabolic dysfunction, oxidative stress, and calcium imbalance, are known to activate multiple intracellular signaling pathways. The subcellular distribution of NF-κB p65 was first examined by immunofluorescence. IAA exposure induced clear nuclear translocation of p65 (Figure 6A).

To obtain a broader understanding of the stress response at the signaling protein level, the expression and activation status of key pathway components were examined by Western blot analysis. IAA exposure led to an increase in the protein level of p38-MAPK and an elevation in p-AKT (Figure 6B). A concurrent rise in NF-κB p65 protein was also detected, consistent with the nuclear translocation observed by immunofluorescence.

The activation of these signaling pathways is expected to influence transcriptional outputs. qRT-PCR analysis revealed that IAA exposure significantly increased the mRNA expression of *IL-6*, a downstream effector of NF-κB signaling, and *p21*, a cell cycle regulatory factor linked to *p38 MAPK* activation (Figure 6C).

### 3.7. Glycational Stress Is an Early Event in IAA-Induced Toxicity

**Figure 7:**
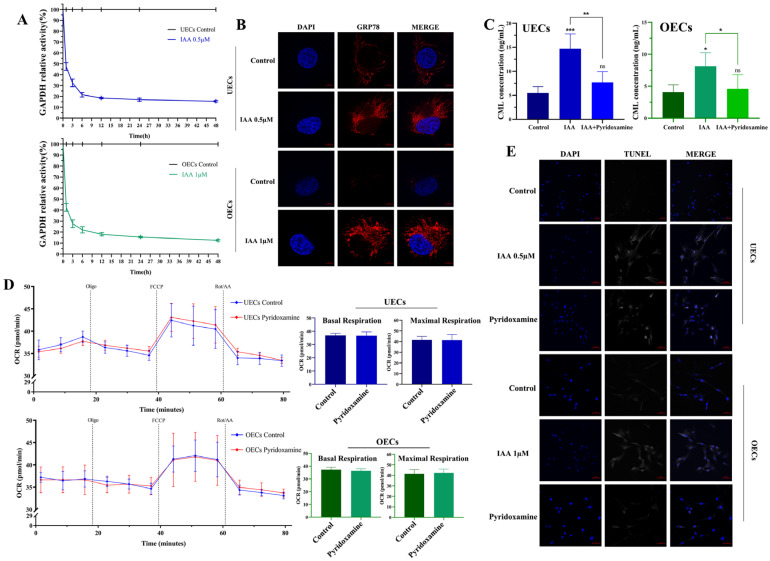
Glycational stress is an upstream driver of IAA-induced toxicity. (**A**) GAPDH enzymatic activity following IAA exposure. (**B**) Immunofluorescence of ER stress marker GRP78 with DAPI-stained nuclei. Scale bar = 5 μm. (**C**) ELISA quantification of AGEs CML in cell lysates. (**D**) Effect of pyridoxamine pre-treatment on IAA-induced mitochondrial OCR. (**E**) Effect of pyridoxamine pre-treatment on IAA-induced TUNEL-positive cells. Scale bar = 50 μm. Among them, n = 3, * *p* < 0.05, ** *p* < 0.01, *** *p* < 0.001.

To investigate the early molecular events responsible for the broad cellular effects induced by IAA, GAPDH enzymatic activity was first assessed. IAA treatment resulted in a significant reduction in GAPDH activity (Figure 7A). Concomitantly, immunofluorescence analysis showed an increase in the ER stress marker GRP78 (Figure 7B).

The accumulation of advanced glycation end products was next examined. ELISA analysis demonstrated that IAA exposure led to an increase in Nε-(carboxymethyl)lysine (CML), and this elevation was reduced when cells were pre-treated with the AGE inhibitor pyridoxamine (Figure 7C). To determine the functional consequences of this glycational stress, rescue experiments were performed. Pyridoxamine pre-treatment improved mitochondrial respiration (Figure 7D) and reduced apoptosis, as evidenced by decreased TUNEL-positive cells (Figure 7E).

## 4. Discussion

In the present study, UECs and OECs were selected as in vitro models. Pigs are increasingly recognized as a highly relevant biomedical model for female reproductive research due to their similarities to humans in terms of reproductive tract anatomy, estrous cycle hormonal regulation, and epithelial remodeling processes. Unlike rodent models, the porcine endometrium undergoes cyclical changes analogous to the human menstrual cycle, making it a superior system for assessing toxicological impacts on epithelial function and fertility potential [24].

IAA, a representative haloacetic acid produced during drinking water chlorination, has attracted considerable attention due to its strong cytotoxicity and potential genotoxic risk. Its widespread detection in diverse aquatic environments raises concerns regarding chronic exposure [25,26]. At the reproductive toxicology level, increasing evidence indicates that IAA can impair female reproductive function. For instance, IAA exposure has been reported to interfere with oocyte maturation in mice and to induce functional impairment in ovarian granulosa cells at elevated concentrations [6,11,27]. Although these findings support the reproductive toxicity of IAA, the initiating molecular events and downstream signaling pathways by which IAA affects epithelial cells within the female reproductive tract remain insufficiently understood.

Epithelial cells serve as the first-line barrier protecting the reproductive tract from environmental insults, and their structural and functional integrity is essential for key physiological processes including fertilization, embryo transport, and implantation [28,29,30]. In this study, primary porcine UECs and OECs were used to investigate the direct cellular effects of IAA. The results demonstrate that through the direct inhibition of GAPDH enzymatic activity via alkylation, IAA leads to pronounced accumulation of AGEs in both UECs and OECs. This glycation-driven metabolic disruption is accompanied by widespread intracellular dysfunction, including impaired energy metabolism, mitochondrial membrane depolarization, redox imbalance, and activation of endoplasmic reticulum stress responses. These interconnected disturbances converge to trigger dysregulation of major stress and inflammatory signaling pathways, such as p38 MAPK and NF-κB, ultimately resulting in cell cycle arrest at the G1/S checkpoint and progression toward apoptosis. Collectively, these findings identify glycation stress as a critical upstream driver linking IAA exposure to mitochondrial dysfunction, oxidative injury, stress pathway activation, and epithelial cell loss in the female reproductive tract.

The transcriptional alterations observed in antioxidant defense and metabolic genes provide further insight into the cellular stress landscape induced by IAA. Expression analysis revealed that genes encoding key components of the redox regulatory network, including NRF2, a master transcription factor governing the antioxidant response, and its downstream targets HMOX1 [31,32], GPX1 [33], CAT [34], SOD1 [35], and SOD2 [36] were significantly upregulated in a cell-type-specific manner following IAA exposure (Figure 2A–F). These genes were selected for analysis based on the well-documented association between IAA exposure and oxidative stress, as IAA is a known inhibitor of glyceraldehyde-3-phosphate dehydrogenase (GAPDH) and an inducer of mitochondrial reactive oxygen species production. Concurrently, the anti-apoptotic gene BCL2 [37], which functions as a critical guardian of mitochondrial outer membrane integrity, was downregulated (Figure 2G), while PKM, encoding a rate-limiting enzyme of glycolysis, showed reduced expression in OECs (Figure 2H). The reciprocal regulation of pro-survival and metabolic genes indicates that IAA disrupts both redox homeostasis and energy metabolism, thereby creating a cellular environment that is incompatible with sustained proliferative capacity. This is consistent with previous reports showing that IAA and related haloacetic acids can induce oxidative stress, metabolic dysfunction, and DNA damage, ultimately constraining cellular renewal capacity [38,39].

Beyond the inhibition of proliferation, the functional consequences of this disruption were reflected in the marked reduction in cell migration (Figure 2I,J), a process essential for epithelial repair following physiological turnover or inflammatory insult. Similar impairments in wound repair capacity have been observed in other epithelial tissues exposed to chlorination byproducts, suggesting that these effects may be systemic rather than tissue-restricted [40,41]. An additional observation is that the suppression of cell migration following IAA exposure was more pronounced in UECs than in OECs (Figure 2J). This disparity may reflect the intrinsically higher metabolic demands and proliferative turnover of the endometrial epithelium, which undergoes cyclic regeneration and remodeling in response to hormonal cues. Accordingly, UECs may be more vulnerable to disruptions in energy metabolism and redox balance induced by IAA, manifesting as a more severe impairment of repair and migratory functions.

An additional observation is the differential sensitivity between epithelial regions of the reproductive tract. UECs displayed notably higher susceptibility to IAA toxicity compared with OECs. Consistently, the suppression of cell migration following IAA exposure was more pronounced in UECs than in OECs (Figure 2J). This disparity may be associated with the intrinsically higher metabolic turnover and hormonally regulated remodeling demands of the endometrial epithelium [42,43]. The endometrium undergoes cyclic proliferation, shedding, and regeneration, requiring robust mitochondrial function and redox balance to support sustained cellular renewal. Disruption of these processes has been linked to implantation failure, impaired decidualization, and abnormal uterine receptivity. Therefore, the increased susceptibility of UECs to IAA exposure both in terms of cytotoxicity and impairment of repair functions suggests that chronic or repeated environmental intake of this contaminant could potentially compromise implantation efficiency and endometrial stability, ultimately influencing reproductive outcomes.

Mitochondrial dysfunction appears to be a central event underlying the cellular response to IAA exposure. In the present work, marked decreases in both basal and maximal mitochondrial respiration (Figure 4A–D) reflect a substantial decline in oxidative phosphorylation capacity, indicating that the metabolic machinery required for sustained epithelial renewal is compromised. Similar reductions in mitochondrial ATP production have been documented in epithelial and hepatic cell models exposed to haloacetic acids, suggesting that mitochondrial impairment represents a conserved toxicity mechanism rather than an isolated cellular response. Reductions in mitochondrial respiratory capacity have been shown to impair not only energy supply, but also disrupt cellular biosynthetic activity, thereby weakening the cell’s ability to maintain structural integrity and repair damage [44,45]. This metabolic insufficiency occurs alongside pronounced disturbance of cellular redox balance. Elevated intracellular ROS levels (Figure 5A) together with a steep reduction in the GSH/GSSG ratio (Figure 4E) indicate an oxidative environment that exceeds the buffering capacity of endogenous antioxidant defenses.

Previous studies on water disinfection byproducts have reported similar oxidative shifts, where ROS accumulation leads to direct oxidative modification of mitochondrial membrane lipids and proteins [46,47,48]. Such oxidative injury contributes to the observed collapse of the mitochondrial membrane potential (Figure 5B), which is widely recognized as a critical event associated with mitochondrial permeability transition and the initiation of downstream apoptotic signaling. Although nuclear translocation of the transcription factor Nrf2 (Figure 5C) suggests an attempt to mount an adaptive antioxidant response, this compensatory mechanism appears insufficient to restore homeostasis under sustained exposure. Importantly, the metabolic and oxidative perturbations are closely accompanied by the disruption of intracellular calcium dynamics. Aberrant cytoplasmic calcium fluctuations and impaired mitochondrial calcium uptake (Figure 4F,G) indicate a breakdown in cross-compartment signaling that coordinates energy demand and supply.

Previous research has demonstrated that calcium coupling between the cytosol and mitochondria is essential for tuning ATP production to cellular workload [49,50]. Therefore, the dysregulation observed here likely amplifies the energy deficit and simultaneously activates calcium-dependent apoptotic pathways. This convergence of metabolic collapse, oxidative overload, and calcium dyshomeostasis positions mitochondrial dysfunction as a central integrative node linking upstream toxic stress to downstream execution of cell death programs.

A growing body of evidence suggests that the earliest molecular disturbances induced by haloacetic acids often originate at the level of protein modification and metabolic enzyme inactivation [38,46,51]. IAA toxicity originates from two primary upstream events: inhibition of GAPDH activity and the accumulation of CML, together establishing a sustained state of glycational stress (Figure 7A,C). Glycation-driven modification of structural and enzymatic proteins has been widely recognized as a critical disruptor of intracellular proteostasis, particularly in epithelial and metabolically active tissues where protein turnover is high [52]. Previous studies in renal and hepatic models have similarly shown that AGE accumulation induces structural misfolding of luminal ER proteins, thereby overwhelming the protein quality control system [53,54]. Consistent with these findings, the pronounced upregulation of the ER stress marker GRP78 observed in this study (Figure 7B) indicates that the endoplasmic reticulum becomes burdened beyond its folding capacity. However, it is important to note that IAA is a broad-spectrum alkylating agent capable of reacting with various thiol-containing proteins and low-molecular-weight thiols such as GSH. Previous studies have shown that iodoacetate can preferentially target GAPDH at low concentrations, and exhibits differential effects compared to iodoacetamide, which is more potent at depleting GSH [55,56]. Therefore, while our data indicate that GAPDH is an early and sensitive target, we acknowledge that other thiol-containing targets may also contribute to the overall toxicity, and the complete molecular hierarchy remains to be fully delineated.

Research in chemical-glycation and diabetic oxidative stress models further support the notion that sustained glycation pressure can drive persistent ER stress and subsequent cellular dysfunction [57,58,59]. Thus, glycational stress emerges here as a central mechanistic axis linking IAA exposure to metabolic dysregulation, proteotoxic injury, and compromised cellular homeostasis. Once proteostasis and metabolic balance are disrupted, a cascading dysregulation of intracellular signaling pathways follows. A defining feature of this response is the simultaneous engagement of survival- and death-associated pathways. The sustained activation of p38 MAPK observed under IAA exposure (Figure 6B) aligns with its well-established role in mediating stress-induced growth arrest and apoptosis, in part through the transcriptional upregulation of *p21* (Figure 6C). Comparable *p38*-driven apoptotic signaling has been documented in epithelial tissues exposed to chlorination byproducts, highlighting a conserved cytotoxic mechanism [60].

In parallel, the significant elevation of phosphorylated AKT (Figure 6B) reflects an intrinsic attempt by the cell to maintain survival and stabilize mitochondrial function. Similar compensatory AKT activation has been reported in oxidative and ER stress models, where transient survival signaling serves to delay, but not prevent, progression toward cell death [61,62]. However, under conditions of sustained mitochondrial damage and redox imbalance, AKT signaling typically fails to fully restore homeostasis, consistent with the outcome observed here. In addition to these survival–death signaling dynamics, the canonical NF-κB pathway is strongly activated, as indicated by nuclear localization of p65 and increased expression of *IL-6* (Figure 6A–C), introducing a prominent inflammatory dimension to the regulation of cell fate.

While NF-κB can confer cytoprotective effects under mild stress, prolonged activation in the context of metabolic dysfunction and oxidative overload is frequently associated with the amplification of inflammatory signaling and the facilitation of apoptotic progression. Similar pro-apoptotic roles of NF-κB mediated inflammation have been described in epithelial responses to environmental toxicants, suggesting that inflammation in this context reinforces rather than counteracts cell death signaling [63,64]. Taken together, the simultaneous engagement of pro-survival AKT signaling and pro-apoptotic p38/NF-κB pathways reflects a fundamental internal conflict between cellular preservation and programmed cell death. The eventual dominance of the apoptotic program demonstrates that the cumulative metabolic, proteostatic, and inflammatory disruptions induced by IAA exceed the adaptive capacity of epithelial cells. As a result, the cellular stress landscape is coherently resolved into a definitive commitment to apoptosis, marking the irreversible loss of epithelial cell viability under IAA exposure.

The accumulation of CML constitutes a defining upstream event in the cellular response to IAA exposure (Figure 7C), indicating the establishment of persistent glycational stress. This process originates partly from the intrinsic alkylating reactivity of IAA, which enables direct covalent modification of cellular proteins, including the glycolytic enzyme GAPDH. Such modification disrupts its catalytic function and compromises glycolytic flux, initiating the formation of AGEs. As a result, the production of AGEs is reinforced through metabolic feedback, creating a self-propagating biochemical environment that gradually destabilizes cellular homeostasis.

Similar feed-forward glycation amplification has been documented in metabolic stress models of endothelial and neuronal injury, suggesting that once established, glycational stress can function as a persistent driver of cellular dysfunction rather than a transient metabolic disturbance [17,18,65]. This mechanistic framework provides a coherent explanation for the downstream outcomes observed under IAA exposure.

AGEs have been widely reported to impair mitochondrial electron transport chain activity, enhance mitochondrial ROS generation, and promote membrane depolarization in epithelial and hepatic systems [66,67]. Studies in renal and pulmonary epithelia similarly demonstrate that CML accumulation interferes with mitochondrial protein turnover and exacerbates oxidative burden, ultimately limiting ATP production and accelerating redox imbalance [68,69]. These well-established effects are consistent with the pronounced reduction in mitochondrial respiratory capacity and GSH/GSSG imbalance observed in the present model. Thus, mitochondrial dysfunction and oxidative stress appear not as secondary consequences, but as direct manifestations of sustained glycational burden initiated by metabolic disruption.

The causal role of glycational stress in driving these outcomes is further supported by the effects of pyridoxamine, a selective inhibitor of AGE formation. Pyridoxamine treatment significantly reduced CML accumulation and concurrently alleviated mitochondrial respiratory impairment and apoptotic progression (Figure 7D,E). Similar protective effects have been reported in models of chemically induced proteotoxic and oxidative injury, where suppression of glycation restored mitochondrial membrane integrity and improved cell survival. Collectively, these results indicate that glycational stress functions as the pivotal upstream driver, directing cells toward mitochondrial failure and eventual loss of viability under IAA exposure.

In conclusion, the present study outlines a mechanistically coherent pathogenic sequence initiated by IAA exposure. Glycational stress, marked by concurrent GAPDH inhibition and accumulation of AGEs such as CML, represents an early and critical disturbance in the pathogenic sequence. This biochemical state progressively disrupts metabolic flux, intensifies oxidative burden, and challenges protein-folding capacity, thereby inducing a sustained cellular stress environment. The convergence of these disturbances on mitochondrial function leads to bioenergetic failure and the dysregulation of survival–death signaling pathways, ultimately resulting in cell cycle arrest and apoptotic commitment.

It is important to acknowledge that the current study has several limitations. The use of primary epithelial cell models provides mechanistic clarity but does not fully reflect the complexity of the reproductive system in vivo, where endocrine signaling, stromal epithelial interactions, immune modulation, and extracellular matrix dynamics collectively influence cellular responses to toxicants.

In addition, the exposure conditions used here represent relatively acute and controlled environments, whereas environmental exposure to IAA in real-world settings is likely to occur at lower concentrations over extended periods. IAA has been detected in source waters and drinking water systems across multiple countries, with reported concentrations typically ranging from 100 ng/L to 2200 ng/L, depending on disinfection practices and source water characteristics (Supplement Figure S1). Notably, comprehensive data on IAA levels specifically in finished drinking water remain sparse, as routine monitoring programs often prioritize regulated haloacetic acids (e.g., dichloroacetic acid, trichloroacetic acid) over iodinated species. Even if one conservatively assumes the highest reported environmental concentration and complete absorption following ingestion, the resulting peak plasma concentration would be orders of magnitude lower. Furthermore, to the best of current knowledge, no biomonitoring data are available regarding IAA concentrations in human blood, urine, or reproductive tract tissues, making direct comparisons between in vitro effective concentrations and in vivo target tissue exposure levels challenging. It is therefore essential to emphasize that the IAA concentrations used herein were selected to establish a robust in vitro platform for dissecting the direct molecular initiating events and downstream signaling cascades triggered by this disinfection byproduct, rather than to replicate chronic, low-dose environmental exposure. In this context, it should also be acknowledged that despite the global reliance on chlorination for drinking water disinfection, clear epidemiological evidence linking IAA exposure specifically to impaired female reproductive health or reduced fertility in human populations is currently lacking. This apparent discrepancy may be attributable to several factors. First, the internal dose of IAA reaching the reproductive tract epithelium under typical environmental exposure conditions is likely to be exceedingly low. Second, intact organisms possess multiple layers of defense, including metabolic detoxification, epithelial barrier integrity, and DNA repair mechanisms, that are absent or saturated in simplified in vitro acute exposure models. Third, chronic, low-dose exposures may elicit subtle, cumulative cellular perturbations that do not manifest as overt reproductive impairment within the timeframes or endpoints typically captured by epidemiological studies. Whether prolonged exposure to sub-cytotoxic IAA levels can, over time, induce cumulative glycative stress and epithelial dysfunction remains an important question for future investigations using long-term, low-dose in vivo models.

Furthermore, the potential contribution of secondary signaling networks, especially the AGE/RAGE axis and its downstream inflammatory amplification, remains to be fully delineated. Given the established role of AGE/RAGE signaling in chronic inflammatory and degenerative pathologies, examining its involvement may uncover additional regulatory dimensions that shape the trajectory from metabolic disruption to epithelial cell loss. With respect to potential interventional strategies, the present findings demonstrate that pyridoxamine, an inhibitor of advanced glycation end-product formation, can attenuate IAA-induced CML accumulation, mitochondrial dysfunction, and apoptosis in vitro. This observation identifies glycation pathways as potential molecular targets for mitigating the reproductive epithelial toxicity of haloacetic acids. Nonetheless, the translational relevance of such interventions remains speculative. Pyridoxamine is a vitamin B6 derivative that has been clinically investigated for diabetic nephropathy, but its efficacy in protecting reproductive tissues from environmental disinfection byproducts has not been evaluated [70,71]. Substantial obstacles remain, including the absence of targeted delivery systems to the female reproductive tract epithelium, the unknown long-term safety profile of sustained pyridoxamine administration, and the practical difficulty of justifying prophylactic intervention against low-level environmental exposures. Future investigations should focus on characterizing endogenous anti-glycation defense mechanisms in reproductive epithelia and evaluating whether dietary or pharmacological strategies can safely enhance resilience to glycative stress without eliciting systemic adverse effects.

In the present study, mechanistic assays were conducted at IAA concentrations corresponding to the IC_50_ values determined for each cell type. At these concentrations, approximately 50% of cells undergo cell death within the 24 h exposure period, and it is acknowledged that some observed alterations could in principle be influenced by ongoing cytotoxic processes. However, several findings support the interpretation that the core mechanistic events described herein precede cell death rather than arising solely as its byproducts [72,73,74]. Glycational stress markers were detectable prior to substantial loss of membrane integrity, and pharmacological inhibition of AGE formation with pyridoxamine attenuated both mitochondrial dysfunction and apoptosis, providing causal evidence that glycative stress drives, rather than merely accompanies, cell death. Future studies employing sub-cytotoxic IAA concentrations will be valuable to further validate this mechanistic cascade under conditions that more closely mimic chronic environmental exposure.

Taken together, several limitations of the present study should be explicitly acknowledged. The use of primary epithelial cell models, while providing mechanistic resolution, does not fully recapitulate the complex in vivo environment of the reproductive tract, where endocrine, stromal, and immune interactions collectively modulate cellular responses. The exposure conditions employed herein were acute and utilized concentrations optimized for mechanistic dissection rather than for replicating chronic, low dose environmental exposure. Furthermore, direct in vivo validation of the proposed glycative stress cascade and its impact on reproductive outcomes remains to be established. Finally, the potential contribution of the AGE/RAGE signaling axis and other secondary networks was not directly interrogated. Future investigations employing long-term, low dose in vivo models and broader pathway analyses will be valuable for extending the present findings.

## 5. Conclusions

**Figure 8:**
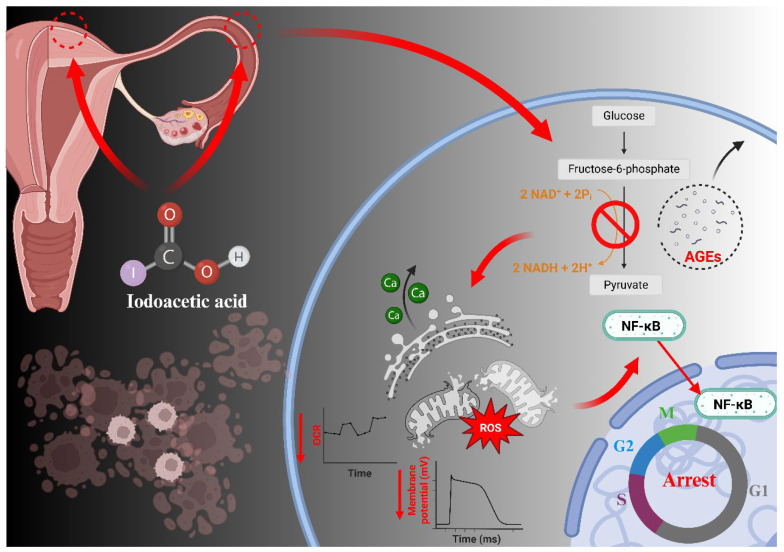
Schematic diagram summarizing the mechanism of IAA-induced epithelial cytotoxicity in the female reproductive system. IAA induces glycational stress via GAPDH inhibition and protein alkylation, leading to AGE accumulation. This initiates ER stress, metabolic dysfunction, oxidative stress, and calcium imbalance, which collectively cause mitochondrial impairment and activate stress and inflammatory signaling pathways, ultimately resulting in G1/S cell cycle arrest and apoptosis.

This study reveals that IAA induces cytotoxicity in UECs and OECs primarily through glycational stress. GAPDH inhibition and subsequent accumulation of AGEs trigger a cascade of cellular disturbances, including metabolic dysfunction, oxidative stress, mitochondrial impairment, calcium imbalance, and activation of stress and inflammatory signaling pathways. These events converge to induce G1/S cell cycle arrest and apoptosis, suggesting that glycational stress plays a central role in epithelial injury. Treatment with the AGE inhibitor pyridoxamine alleviated mitochondrial dysfunction and cell death, supporting the central role of glycational stress (Figure 8). While primary epithelial cell models provide mechanistic insight, in vivo studies are needed to evaluate chronic, low-dose exposure and the potential contribution of AGE/RAGE-mediated inflammation. Overall, this work provides a novel framework for understanding IAA-induced reproductive epithelial toxicity and highlights molecular targets for assessing and mitigating health risks associated with disinfection byproducts.

## Figures and Tables

**Table 1:** Primer sequences.

Gene Name	Forward Primer (5′ → 3′)	Reverse Primer (5′ → 3′)	Accession Number
*GAPDH*	GGTGATGCTGGTGCTGAGTA	AGTGGGAGTTGCTGTTGAAGTC	NM_001206359.1
*NRF2*	AGCAGGACATGGATTTGATTGAC	TTGGTCTTGTGTCTGCTTCTGG	XM_021086339.1
*HMOX1*	TTGACACCAAGGACCAGAGC	GCAGTATCTTGCACCAGGCT	NM_001004027.1
*GPX1*	GTTTCCCGTGCAACCAGTTT	CCACACGCAGGTACAGAGAT	NM_001244489.1
*GPX4*	CATGCGACAGGACCAGTACA	AGGACTGGATCTCGAAGGGT	ENSSSCT00000051893.3
*CAT*	AGAGACTGCCCTGGAGGATA	TTTCCCGAGTCTTTCCAGGT	NM_214301.1
*SOD1*	TGGCCAAGGGTGTGGTT	AGTCACATTGCCCAAGTCTCCA	NM_001190422.1
*SOD2*	AGGAGTTGCTGGAGAAGGAG	CTGGACAAAGTCACGCTTGC	NM_214127.1
*BCL2*	GAGGATTGTGGCCTTCTTTGAG	CCTTCTTTGAGTTCGGTGGG	XM_003127364.5
*FAS*	GGACCCGATGGACTACATGA	CCTTGGTGTTGTCCTTGTCC	AY183428
*PKM*	CGAGGAAAGAGCCCTGATTG	CTGGGTACTGATTATGGCGGA	XM_021096147.1
*IL-6*	GCTTCTGGTGATGACTTCTGTCT	TCCTCAGGAACTCCTTCTGTGAC	JQ839263
*ATF4*	TCTCCAGCGACAAGGCTAT	GTGTCGAACTCTTCTCCGCT	XM_021074313.1
*p21*	CCTGGTGATGTCCGACCTG	GGCGGATTAGGGCTTCCTC	NM_214322.1
*CCND1*	AGGCTGTGCATCTACACCG	CTCCTTCGCACTTCTGCTC	XM_003128712.4

## Data Availability

The original contributions presented in this study are included in the article/Supplementary Materials. Further inquiries can be directed to the corresponding author.

## Supplementary Materials

The following supporting information can be downloaded at: https://www.mdpi.com/article/10.3390/antiox15050545/s1, Figure S1: IAA concentration of tap water in various cities. Data from literature source [2,3,4,5].
