# Supplementary material for: Glycation-Driven Mitochondrial and ER Stress Underlies Iodoacetic Acid-Induced Apoptosis in Porcine Uterus and Oviduct Epithelial Cells
Source: Antioxidants (Basel). 2026 Apr 25;15(5):545. doi: 10.3390/antiox15050545 (PMC13203401; doi:10.3390/antiox15050545)
Supplement: Supplementary file 1 [file antioxidants-15-00545-s001.zip › antioxidants-4251099-supplementary.pdf]

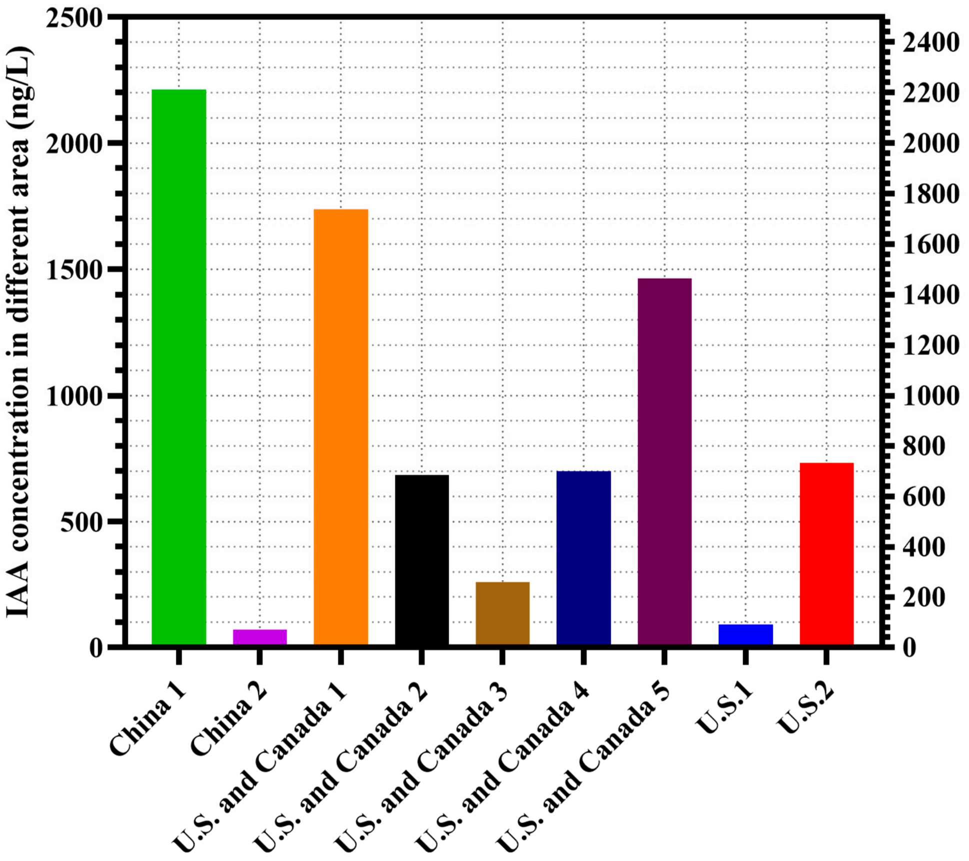

Supplement Figure S1. IAA concentration of tap water in various cities. Data from literature source[1-4]

1. Richardson, S.D.; Fasano, F.; Ellington, J.J.; Crumley, F.G.; Buettner, K.M.; Evans, J.J.; Blount, B.C.; Silva, L.K.; Waite, T.J.; Luther, G.W.; et al. Occurrence and mammalian cell toxicity of iodinated disinfection byproducts in drinking water. *Environ Sci Technol* **2008**, *42*, 8330-8338, doi:10.1021/es801169k.
2. Wei, X.; Wang, S.; Zheng, W.; Wang, X.; Liu, X.; Jiang, S.; Pi, J.; Zheng, Y.; He, G.; Qu, W. Drinking water disinfection byproduct iodoacetic acid induces tumorigenic transformation of NIH3T3 cells. *Environ Sci Technol* **2013**, *47*, 5913-5920, doi:10.1021/es304786b.
3. Allen, J.M.; Cuthbertson, A.A.; Liberatore, H.K.; Kimura, S.Y.; Mantha, A.; Edwards, M.A.; Richardson, S.D. Showering in Flint, MI: Is there a DBP problem? *J Environ Sci (China)* **2017**, *58*, 271-284, doi:10.1016/j.jes.2017.06.009.
4. Pan, Y.; Li, W.; An, H.; Cui, H.; Wang, Y. Formation and occurrence of new polar iodinated disinfection byproducts in drinking water. *Chemosphere* **2016**, *144*, 2312-2320, doi:10.1016/j.chemosphere.2015.11.012.
